# Influence of Fasting during Moult on the Faecal Microbiota of Penguins

**DOI:** 10.1371/journal.pone.0099996

**Published:** 2014-06-30

**Authors:** Meagan L. Dewar, John P. Y. Arnould, Lutz Krause, Phil Trathan, Peter Dann, Stuart C. Smith

**Affiliations:** 1 School of Exercise and Nutritional Sciences, Deakin University, Burwood, Australia; 2 School of Life and Environmental Sciences, Deakin University, Burwood, Australia; 3 QIMR Berghofer Medical Research Institute, Brisbane, Australia; 4 British Antarctic Survey, High Cross, Cambridge, United Kingdom; 5 Phillip Island Nature Parks, Phillip Island, Australia; 6 Centre for Molecular and Medical Research, Deakin University, Burwood, Australia; University of Illinois, United States of America

## Abstract

Many seabirds including penguins are adapted to long periods of fasting, particularly during parts of the reproductive cycle and during moult. However, the influence of fasting on the gastrointestinal (GI) microbiota has not been investigated in seabirds. Therefore, the present study aimed to examine the microbial composition and diversity of the GI microbiota of fasting little (*Eudyptula minor*) and king penguins (*Aptenodytes patagonicus*) penguins during early and late moult. The results from this study indicated that there was little change in the abundance of the major phyla during moult, except for a significant increase in the level of Proteobacteria in king penguins. In king penguins the abundance of Fusobacteria increases from 1.73% during early moult to 33.6% by late moult, whilst the abundance of Proteobacteria (35.7% to 17.2%) and Bacteroidetes (19.5% to 11%) decrease from early to late moult. In little penguins, a decrease in the abundances of Firmicutes (44% to 29%) and an increase in the abundance of Bacteroidetes (11% to 20%) were observed from early to late moult respectively. The results from this study indicate that the microbial composition of both king and little penguins alters during fasting. However, it appears that the microbial composition of king penguins is more affected by fasting than little penguins with the length of fast the most probable cause for this difference.

## Introduction

An intricate and complex relationship exists between a host and its microbiota. The gastrointestinal (GI) microbiota plays a significant role in energy extraction, fat metabolism and storage, production of short chain fatty acids and host adiposity [1]–[4] and has a profound influence on the modulation of host metabolism [5]. GI microbiota have the ability to modify a number of lipids in serum, adipose tissue and in the liver, with drastic effects on triglycerides and phosphatidylcholine. The resident microbiota are also responsible for the production of metabolites that contribute to host fitness and survival [6]. The use of germ free animals has highlighted the importance of GI microbiota on vertebrate hosts. Germ-free animals are not only more susceptible to disease, but also require a greater caloric intake to achieve and maintain a normal body weight [1]. However, when germ free animals are inoculated with the microbiota of conventionally raised hosts, the body fat level of the germ-free host rapidly increases, despite decreased food intake [1], indicating that members of the GI microbiota may modulate fat deposition [7].

Previous studies examining the effect of fasting on the GI microbiota of vertebrates (hamsters, python, mice), have shown that fasting not only alters the composition and diversity of the GI microbiota, but it also influences the host's immune defence [8]–[10] and that interrelationships exist between a host, its microbiota and the hosts nutritional status, diet and physiology [8], [9] These studies however, have concentrated on animals that are relatively inactive during times of nutrient deprivation. Unlike most vertebrates, moulting penguins have to survive long periods of starvation while also coping with increased metabolic demands for feather synthesis and thermoregulation [11], [12]. Therefore, penguins provide an attractive model for investigating the influence of fasting that is associated with the increased metabolic demands.

Many seabirds, including penguins, are adapted to long periods of fasting due to periodic fluctuations in nutrient availability, breeding and moult [13]–[15]. In general, moult occurs post breeding in most penguin species and can last from 2–5 weeks depending upon the species. During moult, penguins replace their entire plumage whilst fasting on land and cannot return to sea because of the consequences of reduced waterproofing and thermal insulation [14], [16]. Throughout moult, penguins must rely on endogenous fat and protein reserves for feather synthesis and nourishment and it is therefore considered to be the most stressful and energetically demanding periods within the penguin life cycle due to increased metabolic demands for feather synthesis and thermoregulation [17]. Many penguin species experience high rates of mortality during and immediately after moult as a consequence of inadequate storage of fat and protein reserves [17]–[19]. Because of its role in host adiposity, immune function and regulation and metabolism, the GI microbiota could potentially influence host health and survival during this stressful period. The king (*Aptenodytes patagonicus*) and little (*Eudyptula minor*) penguin have different moult periods, with moult lasting 2–3 weeks in little penguins, and 5 weeks in kings [20], [21]. Therefore, these two species provide an opportunity to examine the influence of moult and the length of moult on the microbiota. Therefore, the goal of this study was to examine how the GI microbiota changes during moult in king and little penguins.

## Methods

### Ethics Statement

All animal work was conducted according to the national and international guidelines for animal welfare. Animal ethics for this study was approved by Phillip Island Nature Parks Animal Experimental Ethics Committee (#1.2008) (Little penguins) and the British Antarctic Survey Animal Ethics Review Committee (King penguins). All research was carried out under permits issued by the Department of Sustainability and Environment, Victoria (#10004713) and under a permit issued to the British Antarctic by the Government of South Georgia and the South Sandwich Islands.

### Sample Collection

Faecal samples were collected from king (n = 12) and little penguins (n = 9) during early and late moult. King penguins were located at Bird Island, South Georgia an island periodically used by king penguins during moult (54°00′S, 38°03′W), while little penguins were located at the Summerland Peninsula, Phillip Island, Australia (38.4833°S, 145.2333°E).

To obtain faecal samples a sterile Copan E-swab (Copan, Italy) was inserted into the cloaca. The samples was then placed into an amine solution for preservation of the DNA and frozen at −20 during field storage and then stored at −80°C until analysis.

### DNA Extraction and Real Time PCR

DNA was extracted from 0.2 g of faeces using the Qiagen QIAamp DNA Stool Mini Kit (Hilden, Germany) following the manufacturer's standard protocol. The major phyla selected for analysis in this study were selected on the basis of previous studies that had examined the predominant gastrointestinal microbiota of vertebrates [22]–[27], which included Firmicutes, Bacteroidetes, Actinobacteria and Proteobacteria [28]. Quantitative real time PCR was performed on the Stratagene MX3000P as previously described in Dewar et al [28]. Bacterial concentration was determined by comparing the threshold value (Ct. Values) with a standard curve. The standard curve was created by using a serial 10 fold dilution from DNA extracted from a pure culture of *Escherichia coli* ranging from 10^2^–10^10^ CFU/g as per Dewar et al [28].

### Sample Analysis

From the original samples, 4 individuals per species were randomly selected for 16S rRNA pyrosequencing. The 4 purified PCR products from each species were pooled together with the attachment of MID tag barcodes (i.e. Barcode 338R_BC0496 “TCACTTCTCGCT” was attached to all little penguin early moult samples). Samples were then amplified using universal primers Roche adapter A (5′GCC TCC CTC GCG CCA TCA GT-3′) and reverse 338R (5′-CAT GCT GCC TCC CGT AGG AGT-3′) to amplify the V2–V3 region. Following amplification, samples were sequenced on the Roche/454 GS FLX Titanium Genome Sequencer by Engencore (411 University Ridge, Suite A Greenville, SC 29601 USA) according to Fierer et al [29]. All sample preparation and sequencing was performed by Engencore (USA) according to the Roche 454 and Fierer et al [29] protocol. Following sequencing, barcodes were removed using Roche SFF software (Roche Applied Science, Indianapolis, USA).

### Data Processing and Analysis

Quality control, removal of chimera's (Chimera Slayer), clustering of sequences into Operational Taxonomic Units (OTUs) (uclust_ref approach, sequences were aligned to Greengenes database using uclust with 97% sequence-identity cut-off) and taxonomic assignment (RDP-Classifier confidence cut-off  = 0.6) were performed using QIIME (Table S1) [30]. The 16S rRNA sequences reported in this study have been submitted to European Nucleotide Archive (ENA) under accession number ERP001595. Low abundant OTUs were excluded from subsequent analysis, i.e. only those OTUs were included that had >0.005 relative abundance (assigned reads/total number of reads) in at least one sample. Data-mining and statistical analysis was done in Calypso version 3 (http://bioinfo.qimr.edu.au/calypso/).

To determine if there were significant differences between all penguin species for the major phyla for qPCR analysis, a Paired Samples T-test analyses was performed in SPSS with a significance level of p<0.05. Multidimensional scaling (MDS), diversity and cluster analysis were performed on the qPCR data. The separation distance in the MDS plot of samples represents the (dis)similarity of their community profiles.

OTU profiles (relative number of reads assigned to each OTU) of each sample were compared by the Bray-Curtis distance metric (using the vegan R package). The computed Bray-Curtis distances were subsequently used to ordinate the OTU profiles by Principal Coordinates Analysis (PCoA), A Pearson's Correlation Network was performed in Calypso version 3 (http://bioinfo.qimr.edu.au/calypso/). Genera, penguin species and moult state were represented as nodes. Nodes were layout by PCoA based on Pearson's correlation as similarity measure. Pearson's Correlation was calculated on the relative number of reads assigned to each genus. Pearson's correlations >0.5 were visualized as yellow edges, Pearson's correlations <−0.5 as blue edges.

## Results

### Quantitative Real Time PCR

**Figure 1:**
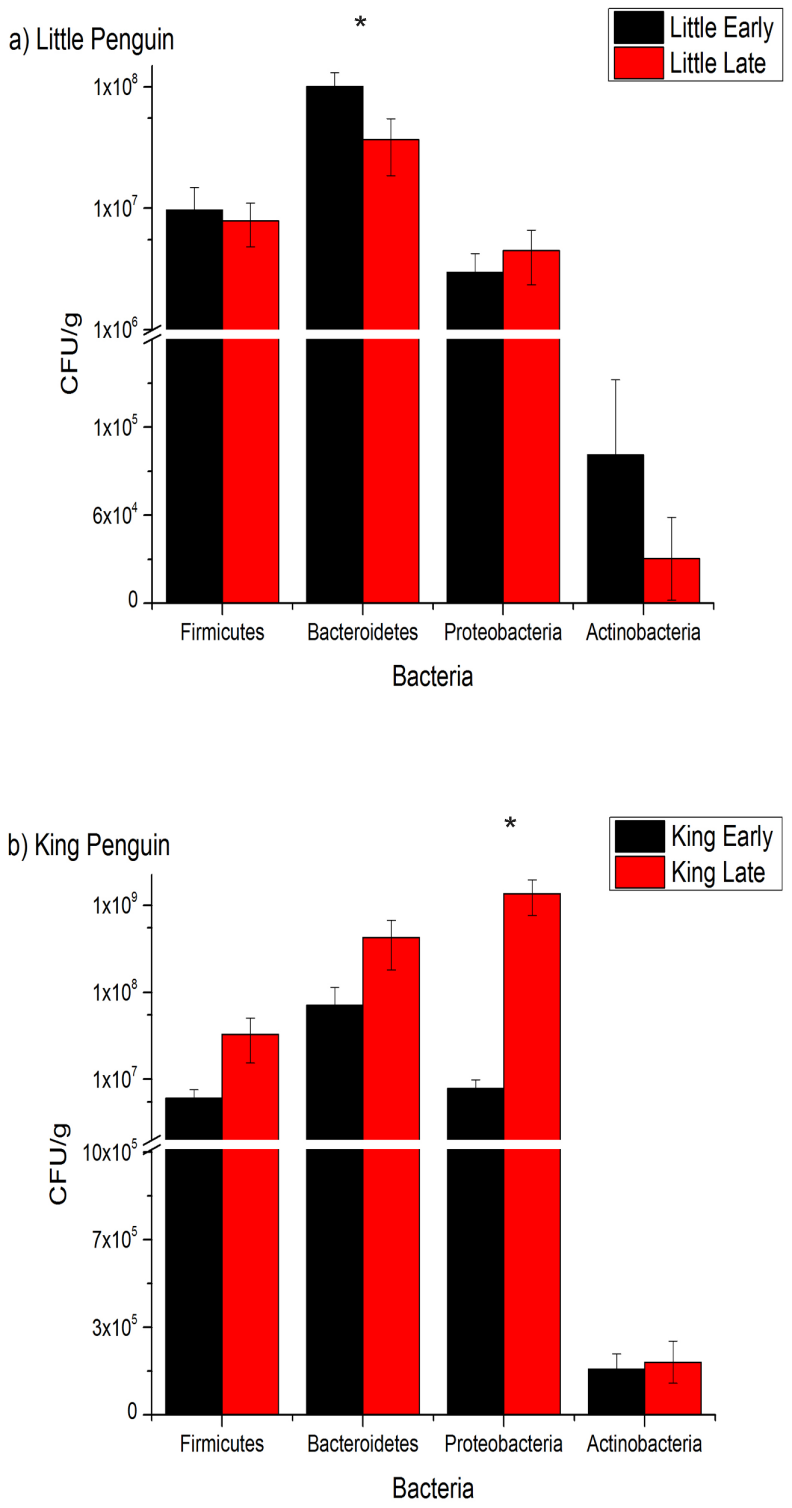
Variation in the abundance of the major phyla; Firmicutes, Bacteroidetes, Proteobacteria and Actinobacteria during moult in king and little penguins. The abundance of the major bacterial phyla was determined by comparing the 435 threshold value (Ct values) with a standard curve.

Quantitative assessments using Quantitative Real Time PCR (qPCR) of the bacterial populations of four major phyla (Firmicutes, Bacteroidetes, Proteobacteria and Actinobacteria) from DNA obtained from faecal samples collected from king (n = 12) and little penguins (n = 9) during early and late moult showed contrasting results. In little penguins, Bacteroidetes was the most abundant phyla followed by Firmicutes, Proteobacteria and Actinobacteria, whilst Bacteroidetes and Proteobacteria, were the most abundant phyla in king penguins. In little penguins a significant decrease in the abundance of Bacteroidetes was observed during moult (p<0.05) (Figure 1). In king penguins a significant increase in the abundance of Proteobacteria was observed during moult (p = .005) (Figure 1).

### Cluster analysis of microbiotas

**Figure 2:**
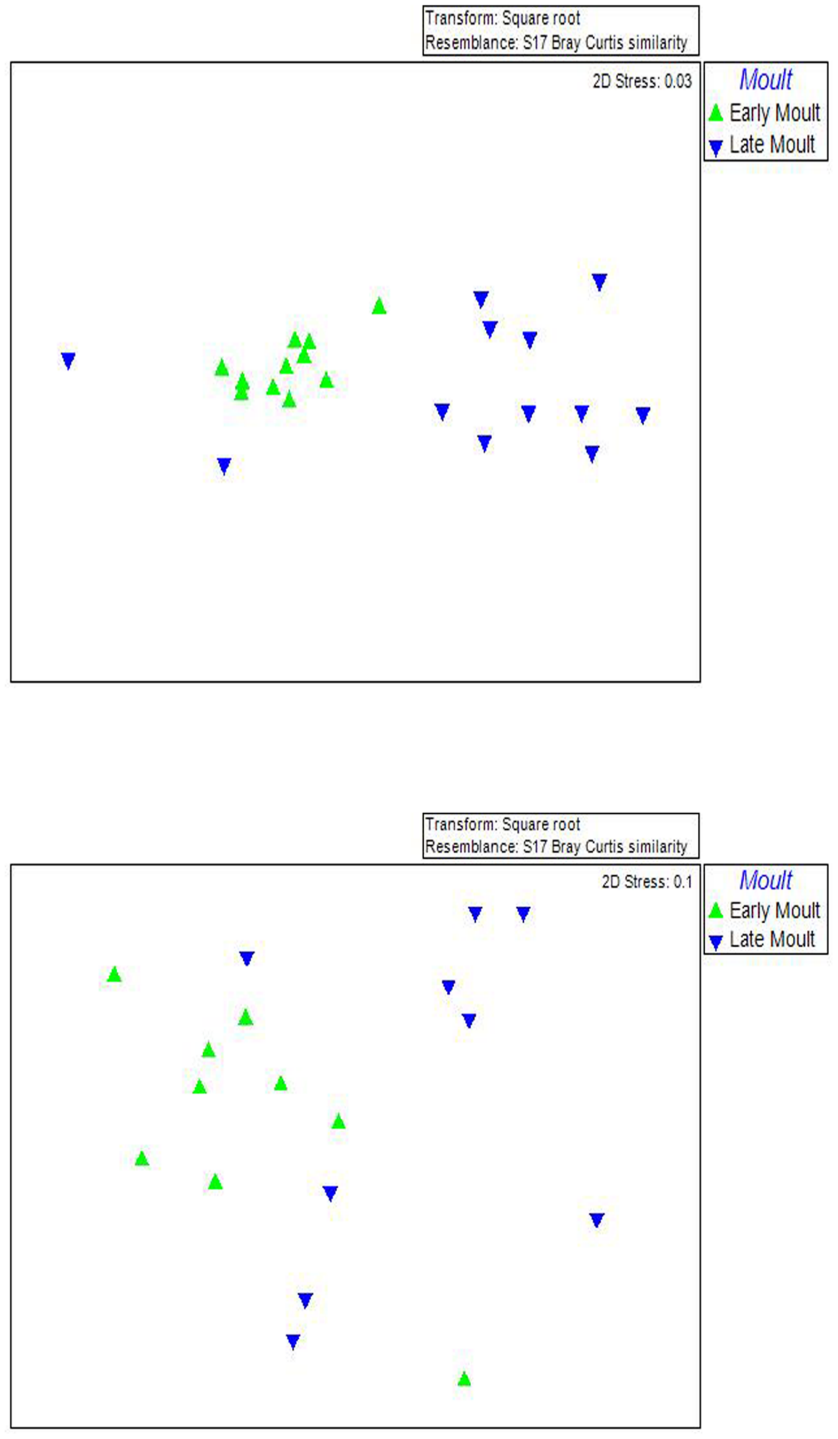
MDS ordination plot of microbiota of fasting king and little penguins based on a Bray-Curtis Similarity matrix of square root transformed qPCR data. Top: MDS analysis of early and late moulting little penguins. Bottom: MDS analysis of early and late moulting king penguins.

The early moult fasting microbial samples cluster together in the MDS Plot, indicating little variation in the microbial composition at the beginning of the moult. However, by late moult, the microbiota of different individuals shows a high variance, indicating the presence of individual variation in response to moult (Figure 2). The cluster analysis also indicates a low level of similarity between the intestinal microbiota of early and late moulting penguins.

**Figure 3:**
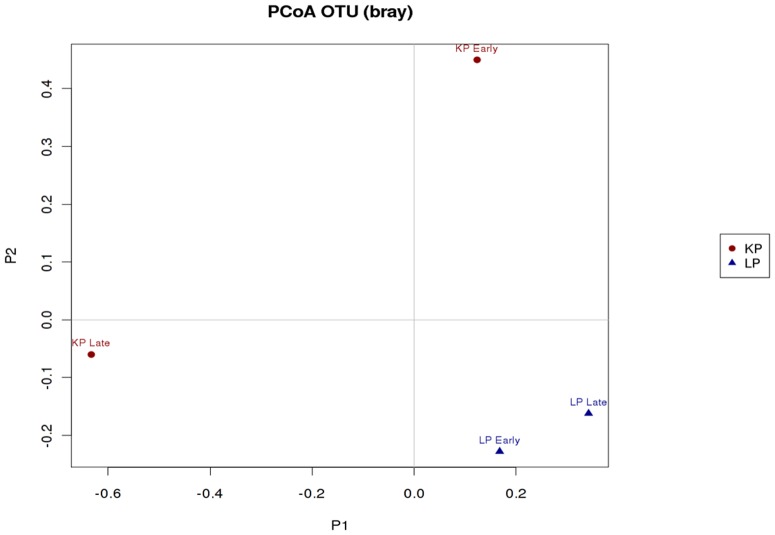
Principal Coordinates Analysis (PCoA) of OTU profiles (Bray-Curtis) of early and late moulting penguins.

The Principal Coordinates Analysis (PCoA) of microbial community (OTU) profiles (Bray-Curtis) shows that there is a high level of dissimilarity between early and late moulting king penguins and a lower level of dissimilarity between early and late moulting little penguins (Figure 3).

### Taxonomic classification of fasting microbiota of penguins

**Figure 4:**
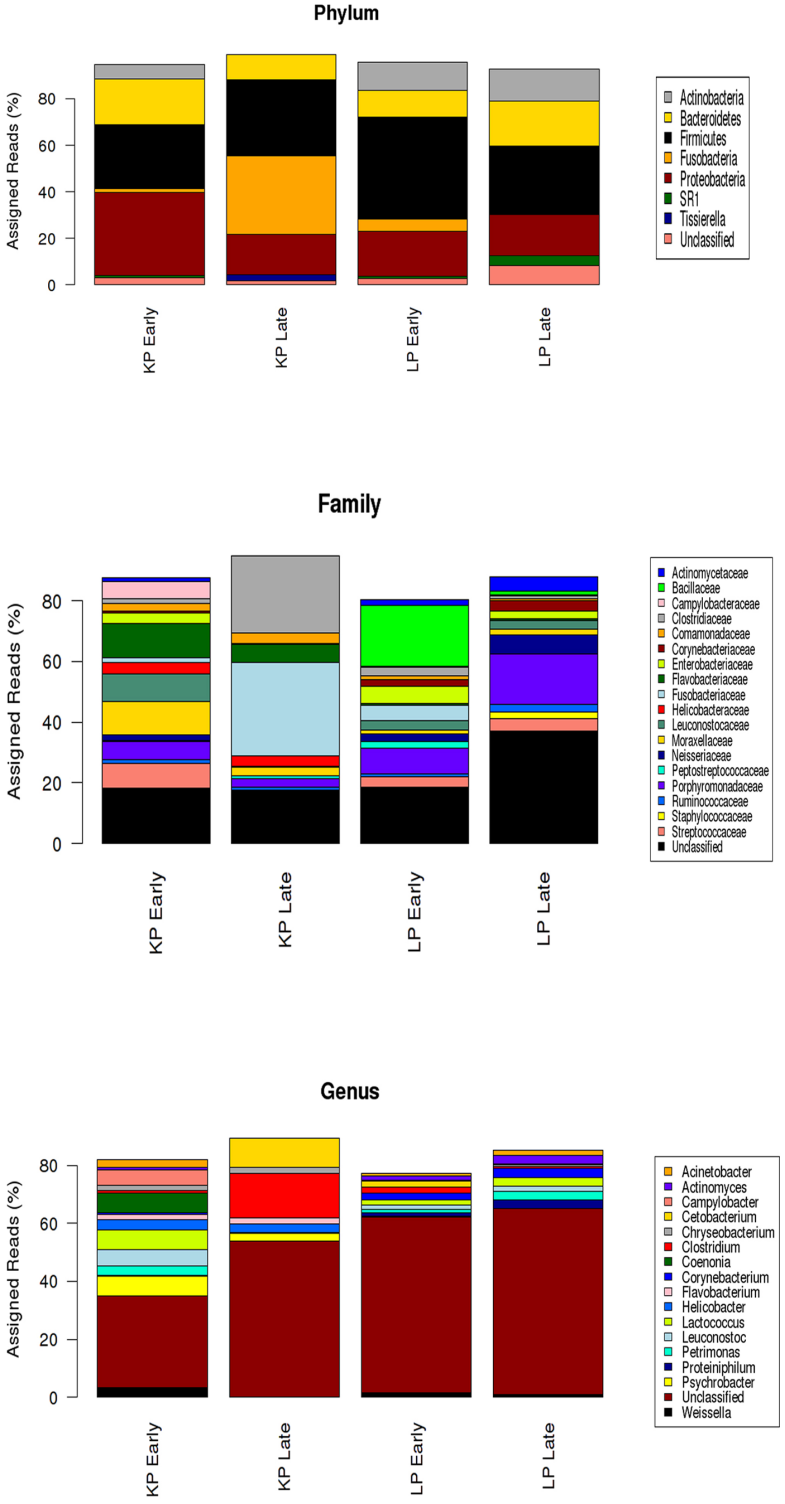
Relative abundance of major taxa in fasting penguin microbiota assayed by 16S high-throughput sequencing. Taxa with relative abundances less than 2% were not included.

A total of 4,986 and 5,856 partial 16S rRNA gene sequences were amplified from faecal samples collected from little penguins during the early and late moult respectively (Figure S1). With 97% sequence similarity a total of 954 and 1,003, phylotypes (Operational Taxonomic Units, OTUs) were identified in little penguins during the early and late moult respectively. Ten bacterial phyla were identified in the little penguin microbiota, with the majority of sequences classified as Firmicutes (29–44%), Proteobacteria (17–19%), Bacteroidetes (11–20%) and Actinobacteria (12–13%). Other less abundant (1–6%) phyla represented were Fusobacteria, Tenericutes, TM7 and SR1. Around 2–8% of 16S amplicons of the little penguin microbiota could not be assigned to any of the known phyla (Figure 4).

In king penguins, a total of 15,151 and 16,749 16S rRNA sequences were amplified from faecal samples collected during the early and late moult respectively (Figure S1). With 97% sequence similarity a total of 2196 and 1551 phylotypes (OTUs) were identified in king penguins during the early and late moult respectively. Six bacterial phyla were identified in the king penguin microbiota, with majority of the sequences classified as Proteobacteria, Firmicutes, Fusobacteria and Bacteroidetes. About 1–3% of 16S sequences of the king penguin microbiota could not be assigned to any of the known phyla (Figure 4).

A high percentage of 16S amplicons from little penguins belongs to uncharacterized families (18–36% of sequences) and genera (61–64%), with less abundant families including Bacilliaceae, Porphyromonadaceae, Enterobacteriaceae, Fusobacteriaceae, Neisseriaceae and Actinomycetaceae (Figure 4). The microbiota of king penguins is dominated by Fusobacteriaceae, Flavobacteriaceae, Clostridiaceae, and Moraxellaceae. At genus level a high number of 16S sequences belong to unknown genera. The most abundant genera were *Clostridium*, *Cetobacterium*, *Psychrobacter Coenonia* and *Lactococcus*.

We observed considerable differences in the penguin microbiota between early moult and late moult. In king penguins the abundance of Fusobacteria increases from 1.73% during early moult to 33.6% by late moult, whilst the abundance of Proteobacteria (35.7% to 17.2%) and Bacteroidetes (19.5% to 11%) decrease from early to late moult. In little penguins, a decrease in the abundances of Firmicutes (44% to 29%) and an increase in the abundance of Bacteroidetes (11% to 20%) were observed from early to late moult respectively.

**Figure 5:**
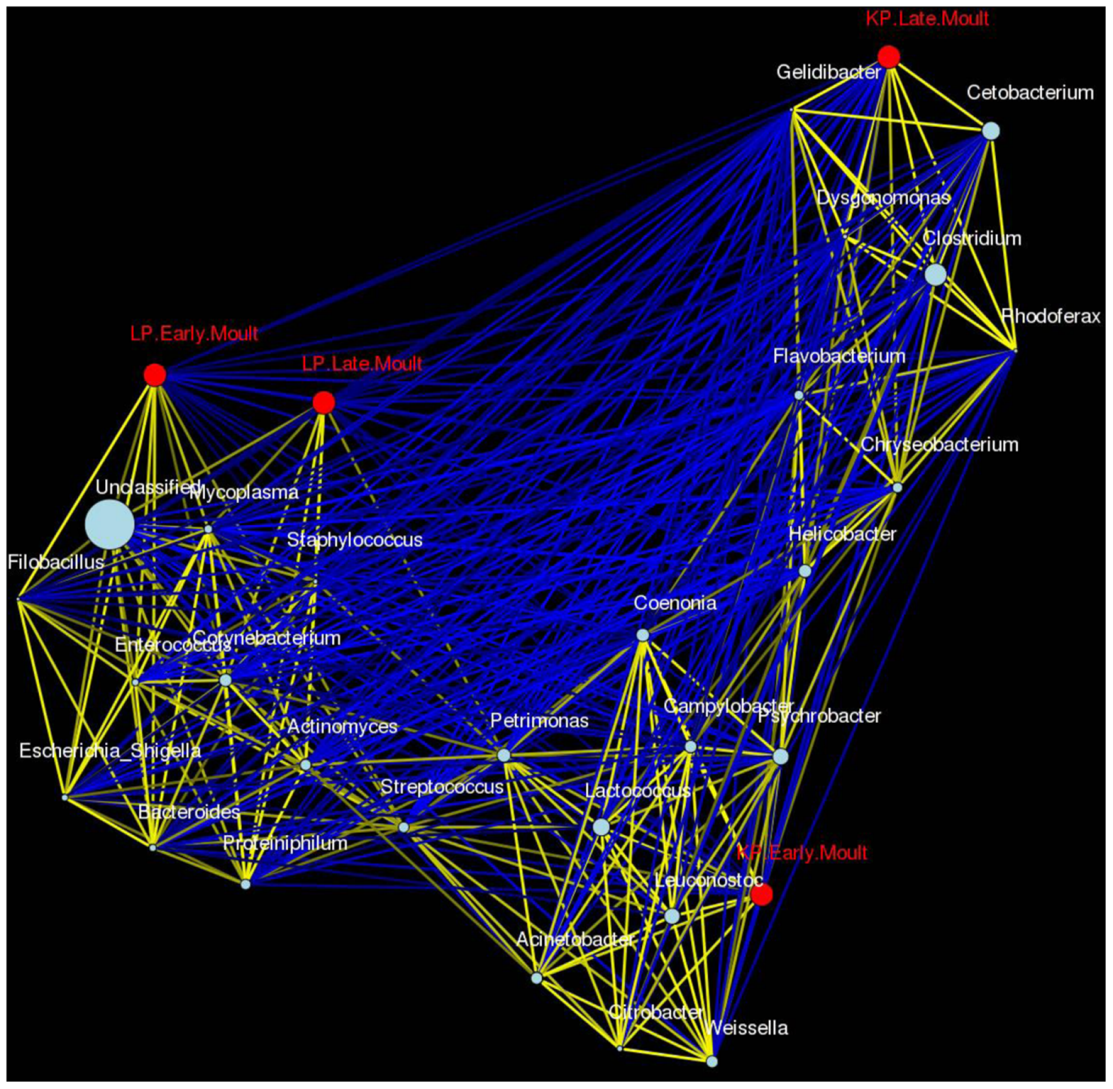
Complex microbe–fasting association network, providing an overview of the associations identified from early and late moulting penguins, visualised in Calypso. The network association identifies the co-occurrence relationships between gut microbial communities of early and late moulting king and little penguins. Each node in the network represents a type of bacteria. Lines connecting nodes (edges) represent significant positive (yellow) or negative (blue) associations as defined by the Pearsons correlation coefficient, with a minimum correlation value of 0.5.

A Complex microbe–fasting association network provides an overview of the associations identified from early and late moulting penguins (Figure 5). The network association identifies the co-occurrence relationships between gut microbial communities of early and late moulting king and little penguins. Genera from the penguin microbiota form three distinct clusters, one associated with early moult king penguins, one with late moult king penguins and one associated with both early and late moult little penguins. In line with our qPCR and PCoA results, the network analysis indicates that the intestinal microbiota of king penguins is considerably different between early and late moult, with the majority of observed bacterial genera being present at only one of the two time points. The microbiota of little penguins on the other hand shows only moderate differences between early and late moult and many bacterial genera were present at both time points. The network analysis further indicates that king and little penguins harbour a clearly distinct, species-specific microbiota on genus level.

## Discussion

Previous studies examining the effect of fasting on the GI microbiota of vertebrate (hamsters, python, mice, termites), have shown that fasting not only alters the composition and diversity of the GI microbiota, and influence the host's immune defences [8]–[10]. Research has also shown that complex interrelationships exist between a host, its GI microbiota and the host's nutritional status, diet and physiological state [8], [9]. These studies however, have concentrated on animals that are relatively inactive during times of nutrient deprivation. Unlike other vertebrates, penguins do not hibernate during times of fasting. In penguins fasting occurs during the breeding season (incubation of eggs and chick brooding) and moult, when penguins replace their entire plumage whist fasting on land. The moulting fasts can last from between 2 and 5 weeks, depending upon species. Therefore, penguins must survive long periods of starvation whilst also coping with increased metabolic demands for feather synthesis and thermoregulation [11], [12]. Penguins provide an attractive model for investigating the influence of fasting that is associated with increased metabolic demands and to date the influence of fasting on the microbiota of any seabird has not been examined. Therefore, to the best of our knowledge, this study is the first to examine the influence of fasting during moult in king and little penguins using qPCR and 16S rRNA pyrosequencing.

### Quantitative Real Time PCR

The quantitative results from the qPCR analysis showed the abundance of Bacteroidetes significantly decreased during moult in little penguins while the abundance of Proteobacteria significantly increased in king penguins. In accordance with other fasting vertebrates, there is a low level of similarity between early and late moult in both penguin species, indicating that moult does alter the microbial composition of both little and king penguins [8]–[10].

### 16S rRNA gene Pyrosequencing

Results obtained from pyrosequencing from the 16S rRNA gene identifying considerable differences in the microbial composition and diversity between early and late moulting penguins. In little penguins, the number of gene sequences were more than three times lower than king penguins with a total of 4,986 rRNA gene sequences from a total of 954 OTU's identified during early moult. By late moult the total number of sequences identified had increased to 5,856 from 1,003 OTU's. In king penguins a total of 15, 151 rRNA gene sequences from a total of 2,196 OTU's were identified during early moult. Although the number of sequences increased during late moult to 16,683 the level of OTU's decreased to 1,551. Similar to other vertebrate species, the most predominant phyla in early moulting penguins were Firmicutes, Bacteroidetes and Proteobacteria [8], [31]–[33]. However, unlike other vertebrates, king penguins were dominated by families Leuconostocaceae, Campylobacteriaceae, Porphyromonadaceae, and Helicobacteraceae, while, little penguins were dominated by Fusobacteriaceae, Bacilliaceae, Porphyromonadaceae and Neisseriaceae.

Increased levels of members from the phyla Firmicutes are associated with increased adiposity, by enhancing energy extraction and through modulation of the genes that regulate fat storage [1], [2], [34]–[37]. Although Firmicutes dominates the microbial composition during early moult in both king and little penguins, it does not constitute a large proportion of the total composition, as in other vertebrates that have large fat stores (i.e. Australian sea lions, polar bears) [32], [38], [39]. This is quite surprising, considering penguins build up large reserves of fat prior to moult. Therefore, one would expect the microbiota of penguins during early moult to have a microbial profile similar to that of other vertebrates that are able to store large fat deposits (i.e. pinnipeds). In accordance with previous studies, a significant shift in the microbial composition was observed in king and little penguins. In Syrian hamsters Sonoyama et al [10] documented that the microbial composition during fed, fasted and hibernating hamsters were all highly dominated by the Phylum Firmicutes. However, the abundance of the class Clostridia was lower in fasted hamsters, while *Akkermansia mucinphila* a mucin degrader were significantly increased in the fasted state. In Burmese pythons, Costello et al [8] documented that the microbial composition during feeding was dominated by *Clostridium, Lactobacillus*, and *Peptostreptococcus*, whilst during fasting, the microbial community was dominated by *Bacteroidetes, Rikenella, Synergistes* and *Akkermansia*. Whereas, Gupta et al [40] documented a 35-fold and 12-fold increases in the levels of Campylobacteriaceae and Helicobacteriaceae in malnourished children when comparing them to healthy children. In this study we saw the complete disappearance of Campylobacteriaceae and Neisseriaceae by late moult and a 52% decline in the abundance of Helicobacteriaceae in king penguins. Whilst in little penguins we see a 60% increase in the level of Neisseriaceae and a 58% decline in the level of Enterobacteriaceae from early to late moult.

Due to the absence of data on the functional role of microbes in penguins we can only infer what impact these changes to the microbiota will have on penguins. Throughout moult the little penguin microbiota was associated with potentially Butyrate producing microbes (*Fusobacteria* and *Clostridia*) [22], [23], known gastrointestinal commensals and known human and veterinary pathogens [24], [25] such as Campylobacteriaceae which has also been associated with disease in penguins [26]. Whilst the king penguin microbiota is dominated by microbes that are associated with butyrate production, chitin degradation, a novel probiotic (*Psychrobacter*) and known gut commensals [22], [23], [27]. Known pathogens such as *Campylobacter*, *Escherichia coli*, and *Helicobacter* also dominate the microbiota during early moult [23]. By late moult, the microbiota is dominated by Fusobacteria, which is a known butyrate producer. In mammals Butyrate is an essential short-chain fatty acid produced in the colon. The main effects butyrate has on the intestinal tract in humans include, influencing ion absorption, cell proliferation and differentiation, immune regulation, and is an important anti-inflammatory agent [41]. In chickens, butyrate supplementation leads to a significant increase in host defence peptide gene expression, enhance antibacterial properties of monocytes against pathogenic bacteria, boost host immunity and increase host adiposity [42]. Therefore the presence of butyrate producing microbes could influence host adiposity levels prior to moult.

## Conclusions

The results from the qPCR and pyrosequencing both indicate that the microbial composition of both king and little penguins alters during fasting. However, it appears that the microbial composition of king penguins is more affected by fasting than little penguins with the length of fast the most probable cause for this difference.

## Supporting Information

Figure S1 Rare fraction Curve for early and late moulting king and little penguins.
(JPG)

Table S1 Taxonomic Assignment of faecal microbiota of early and late moulting king and little penguins.
(XLS)
