# Supplementary figures and images for: Influence of Fasting during Moult on the Faecal Microbiota of Penguins
Source: PLoS One. 2014 Jun 30;9(6):e99996. doi: 10.1371/journal.pone.0099996 (PMC4076183; doi:10.1371/journal.pone.0099996)

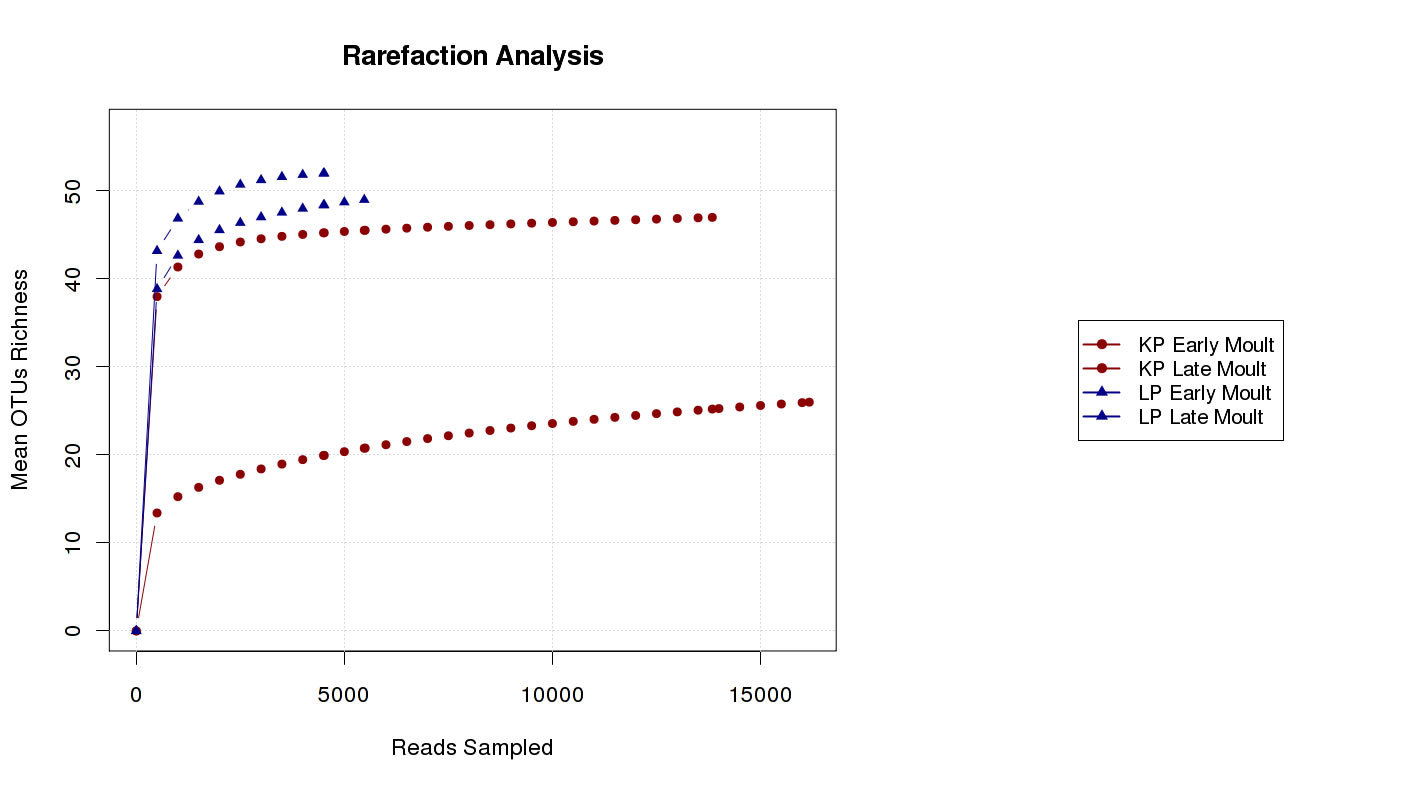

Supplement: Figure S1 — Rare fraction Curve for early and late moulting king and little penguins. (JPG) [file pone.0099996.s001.jpg]
